# The Relation of the State Board of Medical Examiners to the Medical Profession of the State*Read before the Georgia Medical Association, Macon, April 22, 1897.

**Published:** 1897-05

**Authors:** E. R. Anthony

**Affiliations:** Griffin, Ga., Member of the Georgia Board of Examiners


### THE RELATION OF THE STATE BOARD OF MEDICAL EXAMINERS TO THE MEDICAL PROFESSION OF THE STATE.*

By E. R. ANTHONY, M.D., Griffin, Ga.,
Member of the Georgia Board of Examiners.

The crowning glory of the medical profession is its everlasting allegiance to the cause of science and humanity. Its devotion to science and humanity could have been demonstrated in no stronger way than its unceasing and persistent fight, in the last two decades, for a higher standard of medical education.

The men in our profession who should be enshrined in our hearts as objects of undying gratitude are those who have fought hardest for the enactment of a law creating Boards of State Medical Examiners; for the great and broad principle upon which such laws are based, is the protection of the people and the elevation of our profession.

The enactment of such a law had its friends and its foes: the former lauding its advent as a most welcome factor in the elevation of our profession; the latter protesting against it as an infringement upon personal rights and a restriction upon personal liberty. The indifference of legislators to the just demands of our profession for legislation along these lines, and the ignorance of the masses from whom they are selected upon the medical sciences, have retarded and sometimes throttled all legislation which had for its object the promotion and encouragement of medical learning. At last the light of a brighter day has dawned upon us, and our lawmakers have realized the fact that a demand for a higher standard of education for medical men is not only in the interest of the profession, but is a necessity for the public weal. Hence the enactment of the law under which the State Board of Medical Examiners has been created.

So varied are the views held by the public, and by some members of our profession, as to the duties of the Board, and so little

*Read before the Georgia Medical Association, Macon, April 22, 1897.

understood are its powers, that a discussion before this assembly of representative medical men of the "Relation of the State Board of Medical Examiners to the Profession of the State," can but prove beneficial and advantageous. A discussion of the "Relation of the Board to the Profession" can not be made intelligently without necessitating a presentation also of its "Relation to the Applicant for Examination," and the "Duty of the Profession to the Board."

Permit me to say right here, that under the law as it now exists, the State Board of Medical Examiners are not prosecuting officers. No power to deal with the violators of this law has been delegated to the Board. Indeed, to have done so would not only have been without precedent, but absolutely impracticable. This act, no doubt, would be classed by our brethren of the legal profession as a "remedial statute." If it is, we should follow the rule of construction laid down by Blackstone and always construe the act so as to "suppress the mischief and advance the remedy." To "suppress the mischief" in the prosecution of the illegal practitioners is, as it should be, purely a function of the courts; to "advance the remedy" by seeing to it that no incompetent man is licensed to practice, is the duty of the Board. Simply this and nothing more. Every member of the Board in accepting the appointment "incurs a sacred obligation to the profession of the State to exert his best abilities to maintain its dignity and honor; to exalt its standing; and to extend the bounds of its usefulness." This is the great object which the Board should hold steadily in view, and to which it should make every other consideration subservient. To the extent that it advances this object, in the same degree does it discharge its obligation to the State. In carrying out this laudable purpose, it should be governed by wisdom in the protection of the public against unscientific men and methods, justice to the profession in excluding those who would humiliate and break down its claims to rank as one of the learned professions, and moderation in passing upon the merits of the applicants who are knocking timidly at the door of our profession for admission.

It is not within the province of this paper to criticise this law under which the Board of Examiners was created—no one has a right to criticise a law unless he can offer a better in its stead.

That the law has its defects, no one can controvert; that it has its objectionable features, no one can deny; that the Board has not, and cannot have power to deal with violators of this law, is a matter of regret; that the duties of the Board are very simple, we all admit; but repeal this law, defective as it may be: abolish this Board, restricted as its powers and duties are, and you make this State the dumping grounds for quacks and the promised land for impostors. In every country where the medical profession is held in high esteem—"the estimation in which the medical profession is held is always a criterion of the intelligence of a community"—the entrance to the profession is guarded by a Board of Examiners. This then is the essence of the duty of the Board to the profession—to guard its entrance with jealous care and see that no incompetent and unworthy ones enter its sacred precincts.

The past with its diploma mills has bequeathed to us evil results which Examining Boards cannot entirely overcome. It is not within the power of the Board to redress every past wrong; neither can it clear the waters that have so long been fouled by the questionable men who have polluted the stream. To right these wrongs and to correct these evils, we must in a great measure still look to our code of ethics, the great regulator of the doctor's life. One object, dearer to the heart of every true physician than all things else, is the upholding of our code of ethics. "Our profession in a greater measure than either of the other learned professions derives its vigor and its charms from noble sentiments." Abolish the code of ethics and this charm is gone, its vigor is weakened, and our profession degenerates into a trade. "To be ethical means to be a gentleman"—means to be governed by that "etiquette which regulates and controls the conduct of all men who aspire to the moral dignity of gentlemen." A doctor who is not ethical, is a shame to himself, a disgrace to his high calling.

A proposition so plain that it may be accepted as a postulate is, that the environment of the medical profession in our State needs improvement. We believe that the Board will be a great factor in bringing about such improvement, thereby demonstrating the fact that its creation is no failure, its existence is no mistake. We believe that the beneficent workings of this law, if correctly executed, will eventually purify the medical atmosphere of this State and place our profession on a higher plane—too high for the mephitism of quackery to ascend, too high for the malaria of ignorance to reach.

Now as to the "Relation of the Board to the Student."

To be a success as a medical examiner, the examiner most assuredly must possess two requisites: a thorough knowledge of the subject upon which he examines, and absolute impartiality. These are the essentials, the double star the rays of whose light must guide him in all his relations to the applicant for license. The bulk of medical science has increased so wonderfully in the last decade that it is utterly impossible for the medical student to become proficient in the eight leading branches in the short time of three years. If he has become well grounded in the fundamental principles of the leading branches, he has accomplished much. The Board, in conducting its examinations, cannot expect perfection; and it should remember that a "knowledge which falls far short of mastery may be of much practical value." It should formulate questions that are practical tests of the applicant's proficiency and avoid questions that are severe tests of scientific erudition. Such questions do no good except to air the learning of the examiner. And yet these examinations should become more and more severe as the years go by. Not only is there more honor in passing a severe examination, but the one examined knows better how to estimate his acquirements, and then can have that self-confidence that is born alone of knowledge.

The question of preliminary education, what the minimum should be, how this standard should be measured, is preëminently a question in which every member of the medical profession is bound by self-interest and his duty to the public to interest himself. That our profession is more keenly alive to its importance to-day than ever before is a sign full of immense hope for the future. True, under the provisions of this law, it is a question with which the Board has no direct connection; but, despite this fact, we feel that the Board would be recreant to its trust and unfaithful to the high object for which it was created did it not exert all the moral force in its power to secure a higher standard of preliminary education.

Surely we all would welcome such a change, for we can but recognize in it a powerful factor in the work of elevating our profession. The time has come when it is the duty of every friend, and especially every member, of our profession to co-operate and bring about a demand and a plan for a higher preliminary education. A higher preliminary education is not simply an accomplishment, but it exerts a wonderful influence in moulding the life and character of the young man and in preparing him to enter upon the study of the medical sciences. Without it he certainly enters upon the study of medicine at a great disadvantage. There has been urged one weak argument against a compulsory preliminary education for the medical student. That is, that your educated doctor will not locate in the backwoods districts. While it is admitted that learning and education are lost upon a community of uneducated people, we must concede that human life is just as sacred, disease just as difficult of diagnosis, and therapeutics just as hard of application among the unlettered children of poverty as they are among the cultured pets of fortune.

Before discussing the third heading of this paper, "The Duty of the Profession to the Board," I wish to say that the State Medical Association of Georgia can do more than can be expected from any other source to mould public opinion and educate the public mind upon these questions. In bringing about any great reform, we must first touch the great throbbing heart of the public and prepare it for the change. And we must remember that very often, in order to accomplish reforms, it is not only necessary to run counter to the selfish interests of the few, but to fight the customs and superstitions of the many. In the beginning of this paper, we admitted that the law under which the Board was working had its defects, some of which may be remedied by proper amendment. It should be amended so as to require a higher standard of preliminary education, and it should be amended in the matter of dealing with the violators of the law. I would respectfully suggest that the president of this body appoint a committee to discuss and devise the best methods of dealing with the violators of this law, and the best plan of demanding a higher preparatory education; then to formulate their conclusions upon the subject and memorialize the legislature and have the law amended in accordance with these conclusions.

Reciprocity is recognized as a just and wholesome rule of action, and an exchange of equivalents is but another name for justice. The influence for good to the profession of the State in the institution of Medical Examining Boards has already been clearly demonstrated. Then it is nothing but just and proper that the Board should have the sympathies and co-operation of every regular physician in the State. The Board needs your help. There is no member of our profession so high but that he should give it his moral support. The more honors you have gotten from the profession the greater the debt you owe it, and the more binding is your obligation to lend your aid to every movement looking to its advancement. There is no one so humble but that the Board needs his influence. The sun mirrors itself just as faithfully in the tiny dewdrop as it does on the bosom of the boundless ocean. The Board needs your advice, your counsel. It is not infallible, and it is reasonable to suppose that it will make mistakes. In the blaze of that fierce light that always beats upon the administration of a public trust, it expects any mistakes it makes to reveal themselves to the censorious eye of the critic; but it does not regard its critics as enemies, but rather "as torch-bearers welcome for any light they may bring." It expects to have opposition, even from some in the ranks of our profession, but the light thrown out from the "very friction of opposing circumstances" will aid the Board in its work of elevating our calling. We feel that higher medical education is here, and here to stay. Its banner is thrown out to the breezes, and upon its graceful folds, floating high above "against heaven's blue," is inscribed, "Purer ethics, better equipment, grander results." Yes, grander than anything ever done on battlefields, sublimer than aught ever accomplished by eloquent statesmen in senate halls, will be the work of the physician in the cause of science and humanity.

